# Correction: Label free localization of nanoparticles in live cancer cells using spectroscopic microscopy

**DOI:** 10.1039/d3nr90122j

**Published:** 2023-07-12

**Authors:** Graham L. C. Spicer, Luay Almassalha, Ignacio A. Martinez, Ronald Ellis, John E. Chandler, Scott Gladstein, Di Zhang, The-Quyen Nguyen, Seth Feder, Hariharan Subramanian, Roberto de la Rica, Sebastian A. Thompson, Vadim Backman

**Affiliations:** a Department of Chemical and Biological Engineering, Northwestern University Evanston Il 60208 USA; b Department of Biomedical Engineering, Northwestern University Evanston Il 60208 USA v-backman@northwestern.edu; c Departamento de Estructura de la Materia, Física Térmica y Electrónica and GISC, Universidad Complutense de Madrid 28040 Madrid Spain; d Department of Pure and Applied Chemistry, University of Strathclyde, Technology and Innovation Centre 99 George Street Glasgow G1 1RD Scotland UK; e Department of Chemistry, Hunter College New York NY 10065 USA sthompso@hunter.cuny.edu

## Abstract

Correction for ‘Label free localization of nanoparticles in live cancer cells using spectroscopic microscopy’ by Graham L. C. Spicer *et al.*, *Nanoscale*, 2018, **10**, 19125–19130, https://doi.org/10.1039/C8NR07481J.

In addition to the Acknowledgements given by the authors previously (reproduced below), the authors would also like to acknowledge funding from the National Institutes of Health (NIH), through grant #R01CA225002.

Original Acknowledgements:

S. T. acknowledges a PEER/PECRE Travel Grant from WestCHEM. R. R. acknowledges a Ramon y Cajal Contract from Ministerio de Economía, Industria y Competitividad, Agencia estatal de investigacion, Universitat de les Illes Balears, Conselleria d'Innovacio, Recerca i Turisme, and the European Social Fund. I. A. M. acknowledges funding from the Spanish Government through grants TerMic (FIS2014-52486-R) and Contract (FIS2017-83709-R) and from Juan de la Cierva-f program. G. L. C. S. acknowledges funding from NIH RO1 CA173745. This work made use of the EPIC facility of Northwestern University's NUANCE Center, which has received support from the Soft and Hybrid Nanotechnology Experimental (SHyNE) Resource (NSF ECCS-1542205), the MRSEC program (NSF DMR-1720139) at the Materials Research Center, the International Institute for Nanotechnology (IIN), the Keck Foundation, and the State of Illinois, through the IIN.

The Royal Society of Chemistry apologises for these errors and any consequent inconvenience to authors and readers.

## Supplementary Material

